# Revisiting *Jatropha curcas* Monomeric Esterase: A Dienelactone Hydrolase Compatible with the Electrostatic Catapult Model

**DOI:** 10.3390/biom11101486

**Published:** 2021-10-09

**Authors:** Marcos Gustavo Araujo Schwarz, Deborah Antunes, Gabriela Coelho Brêda, Richard Hemmi Valente, Denise Maria Guimarães Freire

**Affiliations:** 1 Laboratório de Genômica Funcional e Bioinformática, Instituto Oswaldo Cruz, Fiocruz, Rio de Janeiro 21040900, Brazil; deborah.santos@fiocruz.br; 2 Laboratório de Microbiologia Molecular e Proteínas, Departamento de Bioquímica, Instituto de Química, Universidade Federal do Rio de Janeiro, Rio de Janeiro 21941909, Brazil; breda.gabriela@gmail.com; 3 Laboratório de Toxinologia, Instituto Oswaldo Cruz, Fiocruz, Rio de Janeiro 21040900, Brazil; richardhemmi@gmail.com; 4 Laboratório de Biotecnologia Microbiana, Departamento de Bioquímica, Instituto de Química, Universidade Federal do Rio de Janeiro, Rio de Janeiro 21941909, Brazil; freire@iq.ufrj.br

**Keywords:** *Jatropha curcas* L., seed, esterase, dienelactone hydrolase

## Abstract

*Jatropha curcas* contains seeds with a high oil content, suitable for biodiesel production. After oil extraction, the remaining mass can be a rich source of enzymes. However, data from the literature describing physicochemical characteristics for a monomeric esterase from the *J. curcas* seed did not fit the electrostatic catapult model for esterases/lipases. We decided to reevaluate this *J. curcas* esterase and extend its characterization to check this apparent discrepancy and gain insights into the enzyme’s potential as a biocatalyst. After anion exchange chromatography and two-dimensional gel electrophoresis, we identified the enzyme as belonging to the dienelactone hydrolase family, characterized by a cysteine as the nucleophile in the catalytic triad. The enzyme displayed a basic optimum hydrolysis pH of 9.0 and an acidic pI range, in contrast to literature data, making it well in line with the electrostatic catapult model. Furthermore, the enzyme showed low hydrolysis activity in an organic solvent-containing medium (isopropanol, acetonitrile, and ethanol), which reverted when recovering in an aqueous reaction mixture. This enzyme can be a valuable tool for hydrolysis reactions of short-chain esters, useful for pharmaceutical intermediates synthesis, due to both its high hydrolytic rate in basic pH and its stability in an organic solvent.

## 1. Introduction

*Jatropha curcas* (physic nut)—a plant belonging to the Euphorbiaceae family—has received attention from the scientific community due to its potential in the biodiesel production field [1]. Biofuel generation can be achieved through different methods such as the alkaline transesterification of the seed oil triglycerides with short-chain alcohol (usually methanol or ethanol) [2]. Such technology is quite advantageous due to the good quality of the produced biodiesel. Furthermore, *J. curcas* can be cultivated on nutrient-poor soils, thus not competing with other crops of agricultural importance [3].

Along with the biodiesel purpose, interest has arisen concerning the remaining material after the oil extraction, known as the press cake, mainly to use it as animal food due to its high protein content [4]. One drawback of such an approach is the presence of different toxic substances in this material, with phorbol esters being the most harmful for animals [5]. That is why non-toxic *J. curcas* strains are used for animal feeding purposes. However, this material can also be a source of several enzymes with interesting features. Such is the case for the previously described *J. curcas* lipase, isolated from the press cake and used in a hybrid (enzymatic/chemical) hydroesterification process for biodiesel production [6].

Several studies focusing on the characterization of proteins/enzymes from the *J. curcas* press cake have been published [7,8]. Furthermore, peptidase [9] and esterases/lipases [10,11,12] have been studied. Esterases and lipases are enzymes belonging to the α/β-hydrolase fold family that generally act on the ester bond of different compounds. Besides hydrolysis, when a low water content medium, such as an alcohol, and an acyl donor are provided, these enzymes can also catalyze esters’ synthesis by different reactions. Esterases/lipases are sometimes organized into subgroups according to their preferred substrate (e.g., triacylglycerol lipases (E.C. 3.1.1.3), phospholipases (E.C. 3.1.1.4), and dienelactone hydrolases (E.C. 3.1.1.45)) [13,14]. Moreover, esterases typically act on water-soluble esters, whereas lipases act at the water/oil interface on “water-insoluble” esters that form micelles in aqueous systems [15]. These enzymes may be used at an industrial scale for several purposes such as for the kinetic racemic resolution of pharmaceutical chiral compounds, as some of these proteins are regio, stereo, and enantioselective catalysts [16].

Regarding *J. curcas* seed esterases, two enzymes with this activity have been described as present in the non-germinated seed: one monomeric (form B) and the other a multimer (form A). In addition, the induction of an enzyme with lipolytic activity occurs during germination. Unlike other esterases/lipases, published data on *J. curcas* monomeric esterase reported pI and optimal hydrolysis pH values that did not fit the widely accepted “electrostatic catapult” model [10], which describes a relevant feature of the esterase/lipase group mechanism of action; in this model, the pI value of the enzyme is usually acidic and therefore lower than the optimal hydrolysis pH value. Furthermore, the model predicts this enzyme group to display higher hydrolytic activity at basic pH due to the negative surface potential surrounding the enzyme active site, repelling one of the final reaction products, namely the carboxylic acid (negatively charged in the neutral and basic pH range) [17,18]. Finally, an important point to consider is that these *J. curcas* enzymes have characteristics of interest to the industry, such as stability and high hydrolytic activity in basic pH conditions [10].

This work aimed to explore the potential of *J. curcas* monomeric esterase as a biocatalyst and better understand how this enzyme could work outside the electrostatic catapult model. Departing from non-germinated seeds, we revisited the enzyme’s purification (albeit partial) and physicochemical characterization and performed protein identification by mass spectrometry. After protein fractionation, the enzyme present in the enriched fraction was identified as belonging to the dienelactone hydrolase family, with an acidic pI and a basic optimum hydrolysis pH; the former contrasts the literature [10] but fit the electrostatic catapult model together with our structural analysis. Moreover, the enzyme is inhibited by divalent cations and is almost inactive in an organic solvent, but not irreversibly. Altogether, our data show that this enzyme may be a valuable tool in hydrolysis reactions with short-chain esters for pharmaceutical intermediates synthesis, for example.

## 2. Materials and Methods

### 2.1. Crude Enzyme Extract Preparation and Ethanol Precipitation

*J. curcas* L. seeds were obtained from DSMM/CATI (Departamento de Sementes, Mudas e Matrizes/Coordenadoria de Assitência Técnica Integral), Águas de Santa Bárbara, São Paulo, Brazil. They were kept under vacuum at 4 °C until processing. Before extraction, dormant seeds were dehulled and albumen was used for acetone powder preparation. Briefly, 200 mL of ice-cold acetone was added to 100 g of albumen and the solid material was ground and further washed with acetone twice. This material was stored at 4 °C until usage.

Proteins were extracted from the seed acetone powder with 0.1 M Tris-HCl pH 7.5 for 1 h. After centrifugation at 16,000× *g* for 20 min, proteins from clarified supernatant were fractionated by ethanol differential precipitation as described by [10]. The first fraction was obtained at 50% of ethanol saturation, followed by centrifugation. Ethanol was added to the remaining supernatant to reach 80% saturation and the material was recovered by centrifugation. Both fractions were resuspended in water and further lyophilized.

### 2.2. Anion Exchange Chromatography

The 50–80% ethanol saturation fraction (50–80% EtOH) was submitted to anion exchange chromatography. The Akta Purifier system (G.E. Healthcare, Uppsala, Sweden) was used at room temperature with the Mono Q 5/50 GL 1 mL column (G.E. Healthcare) and a constant flow rate of 0.5 mL/min. After sample injection, elution was performed with a linear gradient from 0 to 40% of the elution mobile phase in 40-column volumes. The equilibration mobile phase was 20 mM Tris-HCl pH 7.5 and the elution mobile phase composition was the same as the equilibration plus 1 M NaCl. Fractions of 0.5 mL were each automatically collected throughout the elution step and further analyzed.

### 2.3. Enzyme Activity Assays

#### 2.3.1. Esterase

All esterase activity assays were performed using p-nitrophenyl butyrate as a substrate (final concentration of 1 mM) in 0.1 M Tris-HCl buffer (pH 7.5) at room temperature, except when analyzing enzyme chain-length specificity, for which we also used acetate, propionate, laurate, and palmitate p-nitrophenyl esters. Product release was accompanied by spectrophotometry (ThermoFisher Scientific, Woodlands, Singapore) analysis at 348 nm. One international esterase unity (U) was defined as the enzyme amount that released 1 µmol of p-nitrophenol per minute at the assay conditions.

Activity in the presence of divalent ions (Ca^2+^, Co^2+^, Mg^2+^, and Ba^2+^ as their respective chloride salts at the final concentration of 10 mM), solvents (isopropanol, acetonitrile, and ethanol at the final concentrations of 10%, 20%, 40%, and 60% *v*/*v*), and different esterase/peptidase inhibitors (1 mM 4-amidinophenylmethylsulfonyl fluoride (APMSF), 1 mM iodoacetamide, 10 mM ethylenediaminetetraacetic acid (EDTA), 10 µM E-64, 10 µM pepstatin, and 10 mM ortho-phenanthroline) were assessed by adding the different substances into the reaction mixture. For the divalent ions’ assays, enzyme activity was also measured in the presence of each salt with 10 mM EDTA.

We also assessed the esterase activity after sample incubation with 10% and 40% *v*/*v* isopropanol or acetonitrile for 30 min at room temperature, as well as the recovery in an aqueous reaction mixture (0.1 M Tris-HCl buffer pH 7.5).

#### 2.3.2. Peptidase

Peptidase activity was measured using a modified azocasein protocol. Initially, 50 μL of the sample was added to a reaction mixture containing 0.2% (*w*/*v*) azocasein and 10 mM CaCl_2_, obtaining a final volume of 500 µL. After incubation at 37 °C for 1 h, the reaction was stopped by the addition of trichloroacetic acid to a final concentration of 7.5% *w*/*v*. The absorbance reading at 380 nm was performed after the addition of NaOH (final concentration = 0.25 M). Jararhagin, a metalloendopeptidase isolated from *Bothrops jararaca* snake venom [19], was used as a positive control.

### 2.4. Polyacrylamide Gel Electrophoresis (PAGE)

#### 2.4.1. Unidimensional SDS-PAGE

The protein sample complexity was assessed by polyacrylamide gel electrophoresis under denaturant and reducing conditions as described by [20]. Proteins were separated on 15% T gel at a constant voltage of 200 V for 45 min using a Mini-Protean II system (Bio-Rad Laboratories Inc., Hercules, CA, USA). Staining was performed using the Coomassie Blue R-250 protocol [21].

#### 2.4.2. 2D SDS-PAGE

Isoelectrofocusing was performed on strips with immobilized 3.0–10.0 pH gradient (GE Healthcare). Briefly, 130 µg of protein resuspended in a rehydration buffer (7 M urea, 2 M thiourea, and 4% *w*/*v* CHAPS) was added to 150 µL of a solution containing ampholyte 3–10 (10 µL/mL), DeStreak (12 µL/mL), and 5 mM Tris. Focusing parameters were: active rehydration (30 V) for 12 h; 200 V for 1 h; 500 V for 1 h; 1000 V for 1 h; linear gradient from 1000–3500 V over 30 min; and 3500 V until 24,000 Vh were achieved.

After focusing, the strip was treated with 10 mg/mL DTT, followed by 20 mg/mL iodoacetamide incubation, both for 15 min. The second dimension occurred on 15% T SDS-PAGE at constant current (2.5 mA per gel) for 30 min, followed by 5 mA per gel, until the tracking dye left the gel. The gel was stained with the colloidal Coomassie Brilliant Blue G-250 protocol [21].

#### 2.4.3. Esterase Zymography

Samples were loaded in native 15% T PAGE (without SDS and 2-mercaptoethanol) and ran at a constant voltage of 100 V. After this step, the gel was incubated in 0.05% (*w*/*v*) Fast Blue R.R. solution in 0.1 M phosphate buffer (pH 6.2) with 0.02% (*w*/*v*) α-naphthyl acetate until band development.

### 2.5. Protein Digestion, Peptide Extraction, and Mass Spectrometry Analysis

Desired protein spots/bands were excised and in-gel protein digestion was performed based on [22,23] with modifications. Briefly, protein spots/bands were washed three times with 50% (*v*/*v*) acetonitrile in 25 mM ammonium bicarbonate pH 8.0, dehydrated in acetonitrile, and dried in a vacuum centrifuge. For bands derived from unidimensional SDS-PAGE, gel pieces were previously reduced with 65 mM DTT, alkylated with 200 mM iodoacetamide, washed with 100 mM ammonium bicarbonate, and dehydrated with acetonitrile. Gel pieces from both 2D and 1D-SDS-PAGE were rehydrated in 40 mM ammonium bicarbonate containing 20 ng/µL of sequencing grade modified trypsin. Digestion was performed for 16 h at 37 °C. The supernatant was recovered and the remaining peptides were extracted from the gel piece upon 5% (*v*/*v*) formic acid in 50% (*v*/*v*) acetonitrile incubation, followed by sonication. This step was repeated once. The peptide solution was desalted/concentrated using C18 tip-column (ZipTip), following the manufacturer’s protocol.

Desalted peptides were analyzed by MALDI-TOF/TOF (AB SCIEX TOF/TOF 5800) mass spectrometry in reflectron mode. Samples were mixed onto a MALDI plate in a 1:1 ratio with a proper matrix solution (10 mg/mL of α-cyano-4-hydroxycinnamic acid in 50% *v*/*v* acetonitrile and 0.3% *v*/*v* trifluoroacetic acid) according to the dried-droplet methodology. The following peptide mixture was used as an external calibration: Arg-bradykinin (*m*/*z* 904.46), angiotensin I (*m*/*z* 1296.68), Glu-fibrinopeptide B (*m*/*z* 1570.67), ACTH-(1–17) (*m*/*z* 2093.08), and ACTH-(18–39) (*m*/*z* 2465.19). The ten most intense ions from the M.S. analysis were further analyzed in the MS/MS mode, in which fragment-ions were generated by the post-source decay (PSD) process.

Spectral data were analyzed using the PEAKS software (version 5.3), initially by the de novo tool. Error tolerances of 50 ppm and 0.3 Da were used for the precursor and fragment ions, respectively. Semi-tryptic digestion and two missed cleavages were allowed during the search. We also used variable modifications: cysteine (+57.02 Da—carbamidomethylation; +71.04—propionamide) and methionine, histidine, and tryptophan (+15.99—oxidation). Further analysis using the PEAKS DB tool was performed, allowing for variable modifications in cysteine (+57.02 Da). All analyses were done using the non-redundant (N.R.) public NCBI databank into the Eukarya taxon. The false discovery rate was estimated using decoy sequences. Finally, a search was done using the PTM FINDER tool, allowing for variable modification in methionine, histidine, and tryptophan (+15.99 Da—oxidation); serine, threonine, and tyrosine (+79.99 Da—phosphorylation); and N-terminal acetylation of peptides (+42.01 Da), dehydration (−18.01 Da) and deamidation (+0.98 Da). Only results with a false discovery rate (FDR) lower than 1% were reported.

### 2.6. Hydrolysis of Racemic 1,2-O-Isopropylidene Glycerol (IPG) Ester and Diethyl Phenylmalonate

To perform an initial assessment of the *J. curcas* DLH potential in hydrolysis reactions, we tested the enzyme for the racemic resolution of a chiral and a prochiral compound, namely IPG-octanoate (also known as solketal-C8) and diethyl phenylmalonate, respectively. In all reactions, we used the 50–80% EtOH fraction (amount corresponding to 1 U relative to p-nitrophenyl butyrate).

For IPG-octanoate, reaction and analysis were performed as described in [24] with minor modifications. Briefly, hydrolysis was carried out in screw-capped tubes containing 6 mL of 50 mM sodium phosphate buffer at pH 7.0 and 1 U of the enzyme. The reactions were initiated by adding 10 μL of pure IPG-octanoate and the tubes were incubated in a thermostatized reactor (Amersham Biosciences, Freiburg, Germany) (40 °C). Samples were taken at different time points and both enantiomeric excess (ee) and conversion (X) values were determined by gas chromatography on a CHROMPACK CP 9000 (hydrogen flame ionization detector) with a chiral capillary column (Hydrodex-β-6TBDM).

For diethyl phenylmalonate, the reaction was carried out in screw-capped tubes containing 2.5 mL of 100 mM sodium phosphate buffer at pH 7.0 and 1 U of the enzyme. The reactions were initiated by adding the substrate (final concentration of 1 mM) and the tubes were incubated in a thermostatized reactor (40 °C). Samples were taken at different time points and conversion values were determined as described in [25] with minor modifications. Analysis was performed by high-performance liquid chromatography (HPLC) (Spectra Physic SP 100, Stahnsdorf, Germany) using a Kromasil C18 column. Compounds were isocratically eluted (flow rate of 1.0 mL/min) with acetonitrile/20 mM ammonium phosphate (40:60 *v*/*v*) pH 2.6 as the mobile phase and the online UV detection was performed at 225 nm.

### 2.7. Statistical Analysis

Enzymatic activity results are represented as the mean of a set of three independent experiments with standard deviation (S.D.). For statistical analysis, we performed a one-way ANOVA (analysis of variance) test on GraphPad Prism 5 software.

### 2.8. Central Composite Rotational Design (CCRD)

The evaluation of the optimal pH and temperature for catalysis was performed by central composite rotational design (CCRD) with four central points using the 50–80% EtOH fraction. The assay buffer was 20 mM Tris-HCl, adjusted for the desired pH. Esterase activity was measured as previously described. The Table 1 below presents the variables (factors) and levels studied in this experimental design.

### 2.9. Comparative Modeling and Structural Analyses of J. curcas Esterase

We performed comparative modeling using the Swiss-Model server [26] with all default input parameters to derive the three-dimensional structure of *J. curcas* L. esterase B (GenBank accession number KDP24851.1) using the 2.0-angstrom resolution crystal structure of an uncharacterized protein from *Escherichia coli* O157:H7 str. Sakai (PDB ID: 4ZV9) as a template.

The resulting structure was refined using the locPREFMD server [27]. Initial and optimized models were evaluated by the structure assessment tool of the Swiss-Model server. Figures corresponding to the sequence alignment and three-dimensional structures were generated through ALINE software [28] and UCSF Chimera [29] and ChimeraX [30] software, respectively. The electrostatic potential analysis was conducted with the APBS program [31]. The Amber force field charge and radii parameters were assigned using the PDB2PQR server [32], considering the pH values 5.5, 8.0, and 9.5.

## 3. Results

### 3.1. Initial Processing and Chain-Length Specificity

**Figure 1:**
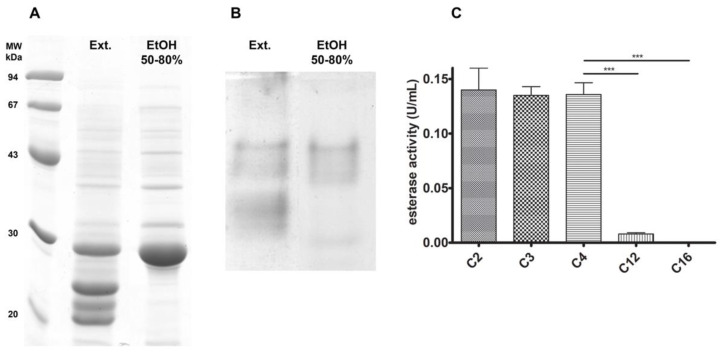
Esterase B enrichment with differential ethanol precipitation. After this fractionation step, the EtOH 50–80% fraction was enriched in a 30 kDa band (**A**) and a single esterase activity region (**B**). Chain specificity assay (**C**) shows that esterase B has greater activity towards short-chain esters, decreasing as chain-length increases. *** *p* < 0.001.

Based on a previous work describing esterase/lipase activities in *J. curcas* seeds [10], we aimed to identify the monomeric protein responsible for one of the esterase activities (named esterase B by those authors), attempting to better understand its features. Repeating the previously described differential ethanol precipitation protocol, we could detect a protein enrichment corresponding to a band around 30 kDa in the EtOH 50–80% fraction for SDS-PAGE (Figure 1A). Aside from that, the esterase zymography PAGE assay also showed enrichment in only one region, in contrast to what was seen in the crude extract (Figure 1B). Enzymatic activity assays with p-nitrophenyl esters confirmed that the EtOH 50–80% protein fraction acted better towards short-chain esters, as assigned by its higher activity towards acetate, propionate, and butyrate ester, decreasing with the chain increase (laurate and palmitate esters) (Figure 1C). These compounds were chosen to perform the initial screening on the esterase chain-length specificity, guiding future substrate choice to assess the *J. curcas* esterase potential as a biocatalyst.

### 3.2. J. curcas L. Esterase B Has an Acidic Isoelectric Point and Belongs to the Dienelactone Hydrolase (DLH) Family

**Figure 2:**
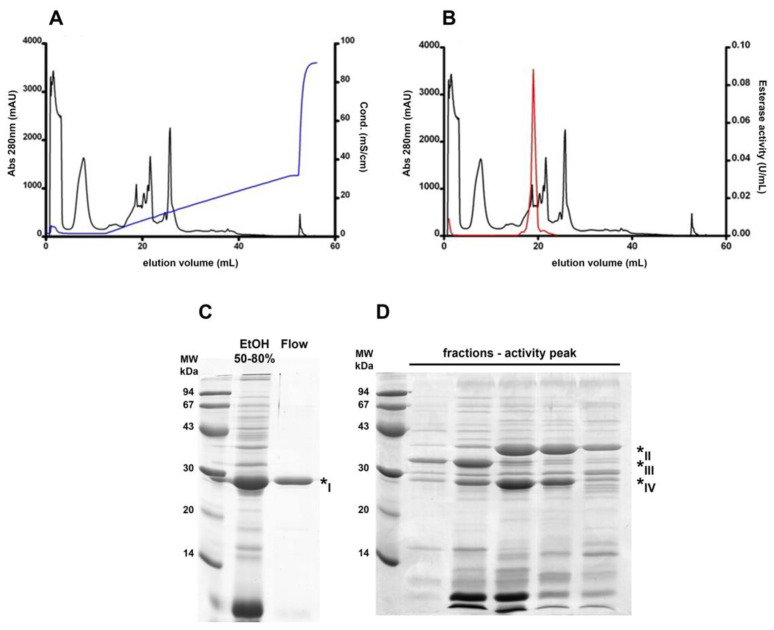
Different esterase B behavior in an anion exchange chromatography. Online absorbance (280 nm) detection was performed (black curves in (**A**,**B**)) and each sample was further assayed for esterase activity (red curve in (**B**)). Resin-bound protein elution was performed by a linear gradient of elution buffer (blue curve in (**A**)). SDS PAGE analysis shows a single observable band in the flow-through fraction (**C**) and enrichment in three bands around the 30 kDa marker in the activity peak fractions (**D**). Band numbering corresponds to the MS protein identification data from Table 2.

Esterase B was previously characterized as a monomeric protein with higher esterase activity towards short-chain esters and pI 9.0, and with an optimum pH around 7.5 [10]. With these features, we designed a chromatographic step aimed at a fraction enriched in this protein. Therefore, an anion exchange chromatography was performed with a pH 7.5 buffer. In this situation, most protein molecules should have an overall negative charge, binding to the positively charged resin; however, esterase B would be expected to exhibit an opposite behavior, eluting in the flow-through. As seen in Figure 2A, there were two prominent peaks in the unbound protein fraction when assessing absorbance at 280 nm and these pooled fractions were enriched in a 30 kDa protein (Figure 2C). Nevertheless, Figure 2B shows that the esterase activity was negligible in the flow-through compared to the activity peak detected along the buffer gradient (as a result, this peak was named “activity peak”). These gradient-eluted fractions were still complex in their protein content, even though they were enriched in three bands around the 30 kDa marker (Figure 2D).

Protein identification by mass spectrometry analysis of these enriched bands, as shown in Table 2, revealed that the main component of the major band within the chromatography flow-through was curcin (* I), a common and highly abundant protein found in the *J. curcas* seed. The three bands within the activity peak were identified as malate dehydrogenase (* II), lactoylglutathione lyase (* III), and a putative carboxymethylenebutenolidase (* IV); bands * III and * IV had relative molecular masses closer to the previously identified 30 kDa for esterase B.

**Figure 3:**
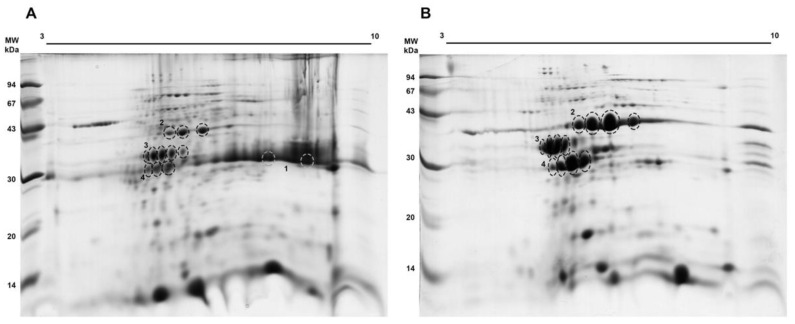
Proteomic profile of the EtOH 50–80% (**A**) and activity peak (**B**) fractions. The indicated spots were processed and peptides were submitted to mass spectrometry for protein identification (Table 2).

We performed a 2D electrophoretic analysis to better physicochemically characterize these samples. We used both the EtOH 50–80% fraction and the higher esterase activity fraction after the anion exchange chromatography. As seen in Figure 3A, in the EtOH 50–80% fraction, we could identify spots corresponding to malate dehydrogenase (* 2), lactoylglutathione lyase (* 3), and the putative carboxymethylenebutenolidase (* 4), the last one having a similar molecular mass as the protein streak towards basic pH corresponding to curcin (* 1), already known to be a basic protein in the *J. curcas* seed proteome [33]. We observed that this curcin streak was no longer detected among the proteins within the activity peak after the chromatographic step (Figure 3B). Spot trains seen for regions indicated as * 2, * 3, and * 4, ranging from pH 5.0–7.0, indicated that malate dehydrogenase, lactoylglutathione lyase, and carboxymethylenebutenolidase (all proteins enriched in the activity peak; Figure 2D) are present as multiple isoforms.

**Figure 4:**
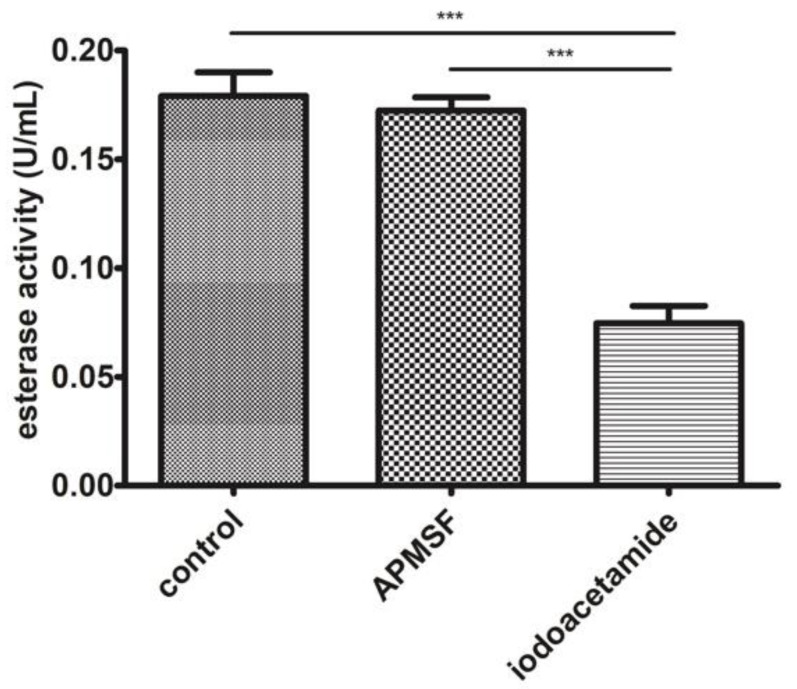
Esterase activity analysis in the EtOH 50–80% fraction with serine (APMSF) and cysteine (iodoacetamide) esterase inhibitors. *** *p* < 0.001.

Among the three identified proteins, carboxymethylenebutenolidase has been characterized as harboring esterase activity. It is also known that cysteine is used within its catalytic triad as the nucleophile during the catalysis cycle. As seen in Figure 4, APMSF did not affect the EtOH 50–80% fraction esterase activity. In contrast, iodoacetamide, an alkylating compound, decreased about 60% of the original activity compared to the control sample.

**Figure 5:**
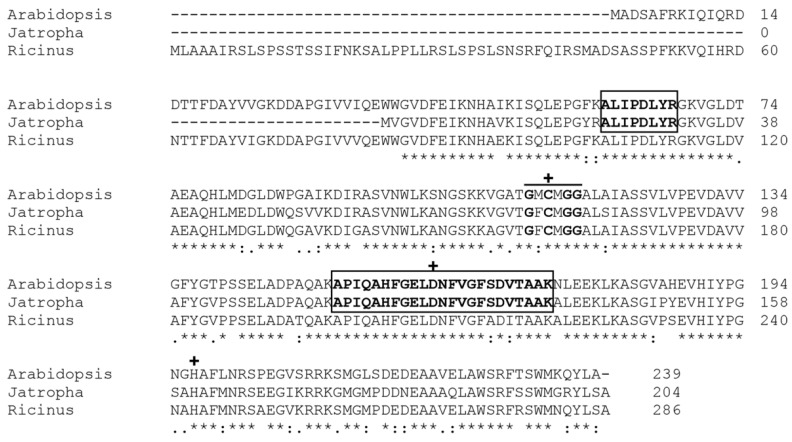
Alignment analysis of *A. thaliana* carboxymethylenebutenolidase and its homologs in *J. curcas* and *R. communis*. Both peptides identified during the mass spectrometry analysis are shown within rectangles. The typical dienalactone hydrolase pentapeptide (GxCxGG) is highlighted, as well as the active site residues Cys-78, Asp-126, and His-161 (marked with +). (*) Conserved sites, (:) sites with conservative replacement, (.) sites with semiconservative replacement.

As seen in Table 2, the identified carboxymethylenebutenolidase (belonging to the dienelactone hydrolase (DLH) family) was from the *Arabidopsis thaliana* proteome. We performed a Blatsp search to identify its homolog within the *J. curcas* context. In Figure 5, we can observe that the sequence from *J. curcas* (GenBank accession number KDP24851.1) shares a high similarity with both the *A. thaliana* (Q8LDC7) and *Ricinus communis* (XP_002524839.1) protein sequences. The catalytic triad (Cys-78, Asp-126, and His-161) was also identified by comparison with the one from *Pseudomonas knackmussii* dienalactone hydrolase (PDB: 1DIN) [34]. We could assess the *J. curcas* protein theoretical pI and molecular mass as 5.74 kDa and 22.09 kDa, respectively, using Expasy tools (https://web.expasy.org/compute_pi/) (accessed on 13 January 2021).

### 3.3. J. curcas L. Esterase B Activity Increases in Basic pH, Corroborating “the Electrostatic Catapult” Model

**Figure 6:**
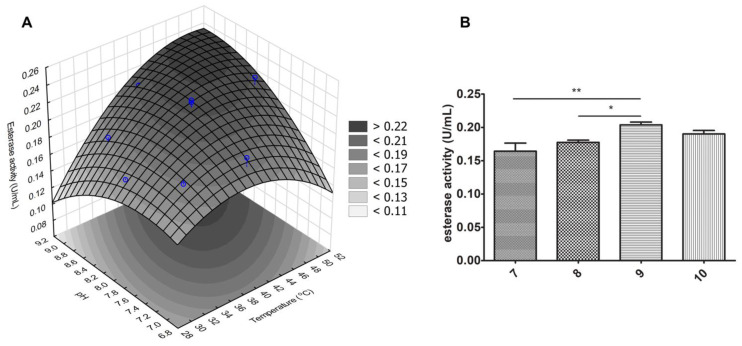
Temperature and pH effects in esterase activity of *J. curcas* esterase B. (**A**) Assessment of their effects were done with a central composite rotational design. Higher esterase activity was observed with higher temperatures and pH values, as observed in (**B**). * *p* < 0.05 and ** *p* < 0.01.

To better assess the effect of both temperature and pH (factors) in the esterase activity (dependent variable, DV), we performed an experimental design (DOE) to obtain the model describing the relationship between these analyzed variables. As seen in Table 3, only temperature, both in linear and quadratic terms, was significant (*p*-value < 0.05) in describing esterase activity variation within the constructed model. Table 4 and Figure 6A show that increasing temperature within the studied range (30 to 50 °C) had an overall positive effect on the enzymatic activity (model optimum temperature of 48 °C). Notwithstanding the non-significance within the model, pH also affected this dependent variable, as observed in Figure 6B, in which higher activities were detected for more basic buffers (pH 9.0–10.0).

### 3.4. Different pH Values Alter the Electrostatic Potential of the J. curcas Esterase B Catalytic Site

**Figure 7:**
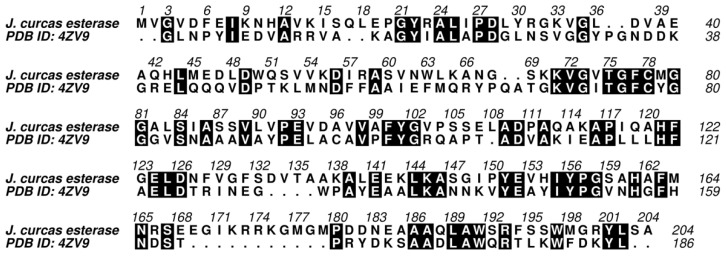
Pairwise sequence alignment employed for the comparative modeling of the *J. curcas* esterase based on the structure of the uncharacterized protein from *E. coli* O157:H7 str. Sakai (PDB ID: 4ZV9). The sequences have 37% identity and 89% coverage. The active site residues—Cys-78, Asp-126, and His-161- are conserved between target and template. Black filled positions in the sequence alignment represent identical residues. Points represent gaps.

To gain insight into the influence of pH on the *J. curcas* esterase catalytic site, we built a tridimensional model based on the crystallographic structure of an uncharacterized protein from *Escherichia coli* displaying 37% and 89% sequence identity and coverage, respectively (Figure 7). The model was refined (method section) and its structural quality was assessed before and after the optimization procedure (Table 5). The optimization helped to improve the quality of the model, making it compatible with the template evaluation. The MolProbity score was better for the refined model than for the template, with values of 0.88 and 0.98, respectively. A score equal to zero represents the structure having no stereochemical problems. The model’s QMEAN had a lower score than the template (−2.39 and 0.02, respectively). However, this value was compatible with high-quality models, making it suitable for subsequent analysis.

**Figure 8:**
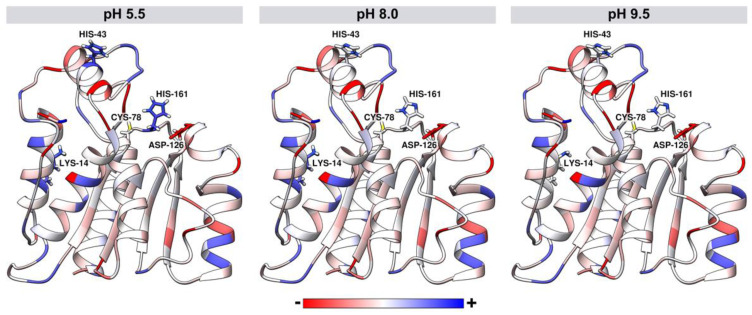
Tridimensional model of *J. curcas* esterase B developed by comparative modeling. Structures are colored according to the atomic charge for different pH values: 5.5, 8.0, and 9.5. Sticks represent the catalytic triad residues (Cys-78, Asp-126, and His-161) and the residues that had protonation change according to the pH values (Lys-14 and His-43).

The pKa values of ionizable groups of *J. curcas* esterase were assessed for pH values 5.5, 8.0, and 9.5. The pH influenced the total protein charge with values of −2.0, −4.0, and −5.0 for pH 5.5, 8.0, and 9.5, respectively. For all pH values, Cys-78 and Asp-126 of the catalytic triad are neutral and negatively charged, respectively. However, at the acidic pH of 5.5, the His-161 of the catalytic site is positively charged, while at both basic pH values (8.0 and 9.5), histidine is neutral (Figure 8). Other differences were also found in residues more distant from the catalytic triad, such as His-43 positively charged in acidic pH and neutral in basic pH values. Lys-14 is neutral at pH 9.5 and positively charged at pH 5.5 and 8.0 (Figure 8).

**Figure 9:**
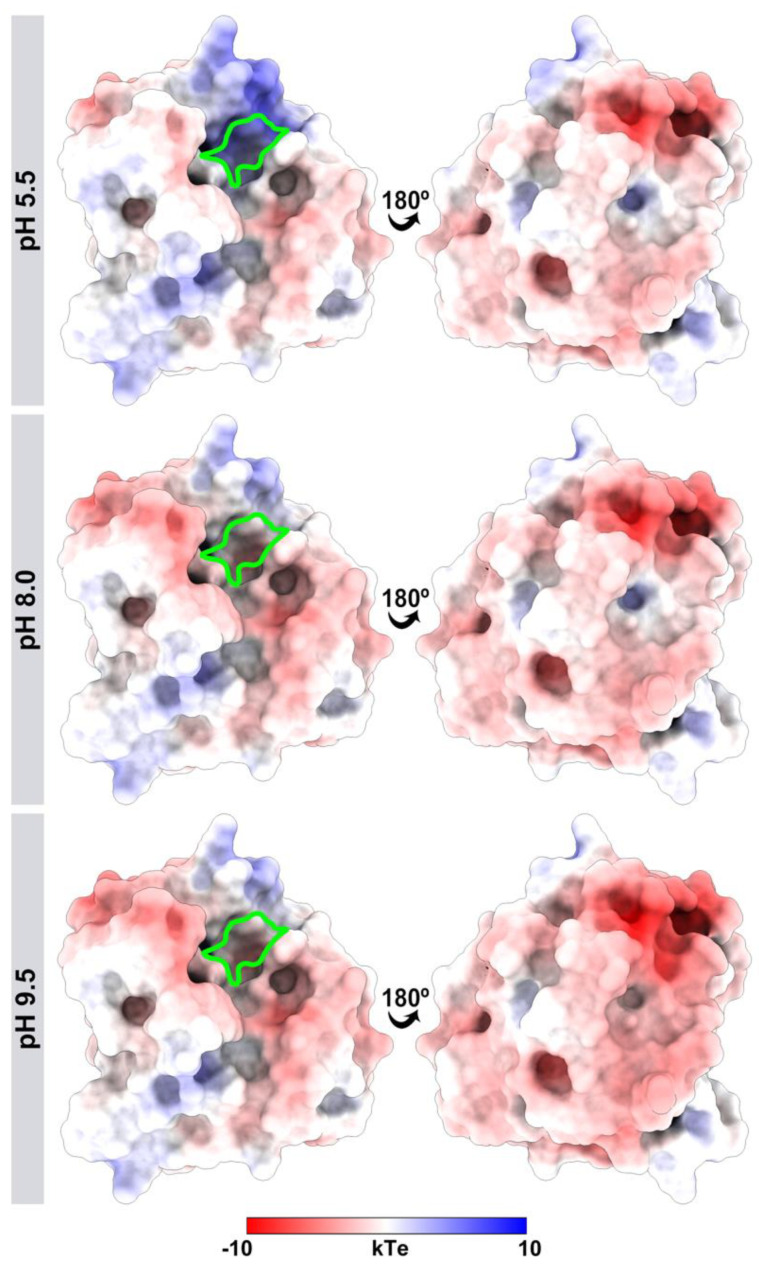
Differences in the electrostatic potential of *J. curcas* esterase B at different pH values (5.5, 8.0, and 9.5). The catalytic triad region (Cys-78, Asp-126, and His-161) is highlighted in green. The molecular surface is colored according to the electrostatic potential, where red, white, and blue correspond to acidic, neutral, and basic potentials.

His-161 altered the surface of the electrostatic potential in the catalytic triad (Figure 9). At pH 5.5, positively charged histidine made the site with basic potential. In contrast, the neutral histidine in the basic pH values left the region with neutral-basic potentials.

### 3.5. J. curcas L. Esterase B Has No Proteolytic Activity and Divalent Ions Inhibit the Enzyme

**Figure 10:**
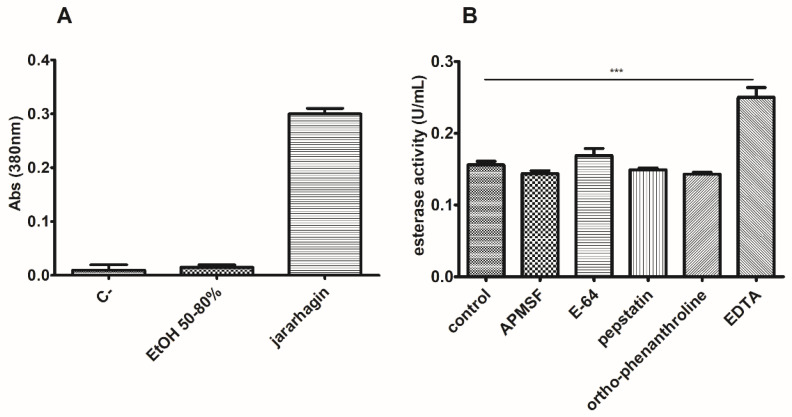
Esterase activity is not due to proteolytic activity. The azocasein assay (**A**) could not detect significant peptidase activity in the EtOH 50–80% fraction. (**B**) Enzymatic analysis in the presence of different peptidase inhibitors corroborated the former finding. Noticeably, the esterase activity of this fraction was enhanced by EDTA addition. *** *p* < 0.001.

We also assessed the peptidase activity for our working sample (EtOH 50–80% fraction). We used an azocasein assay and also tested esterase activity in the presence of known peptidase inhibitors. In Figure 10, we can observe that there was no detectable peptidase activity and that the inhibitors did not decrease esterase activity. However, when assaying with EDTA, a significant increase in esterase activity was detected, about 160% of the control sample value.

**Figure 11:**
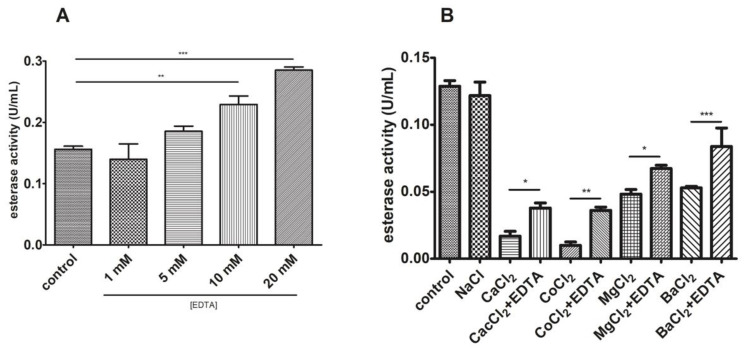
Divalent cations inhibit esterase activity. (**A**) A dose-response relationship was observed between esterase activity and EDTA. (**B**) Assaying with different divalent cations corroborated this and the EDTA addition slightly reduced this impact. * *p* < 0.05, ** *p* < 0.01, and *** *p* < 0.001.

To further characterize this phenomenon with EDTA, a dose-response experiment was performed. As observed in Figure 11A, an increase in EDTA concentration within the reaction mixture enhanced esterase activity in a dose-response relationship. We also tested activity in the presence of different cations alone and with the EDTA addition. Figure 11B shows that esterase activity was drastically reduced in the presence of several divalent cations (reduction with Co^2+^ > Ca^2+^ > Mg^2+^ > Ba^2+^) and that this could be partially reverted when EDTA was also added.

### 3.6. J. curcas L. Esterase B Has Low Activity in the Presence of Different Solvents

**Figure 12:**
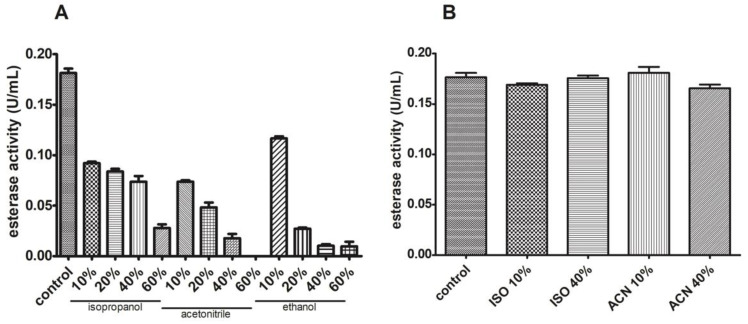
Esterase activity is reversibly lowered in the presence of organic solvents. Esterase activity decreased when isopropanol, acetonitrile, and ethanol were added (**A**) but sample reconstitution in an aqueous reaction mixture reverses this (**B**).

Esterase activity was assessed in the presence of different organic solvents in the reaction mixture. We have chosen these concentrations to screen different scenarios in which this enzyme could be used as a biocatalyst (low to high organic solvent concentration). For all cases, a severe significant reduction was observed, with a 100% inhibition for 60% (*v*/*v*) acetonitrile and a 40% decrease for 10% (*v*/*v*) ethanol, both compared to the control sample (Figure 12A). We also tested the activity after sample incubation with the organic solvent and recovery in an aqueous reaction buffer. In Figure 12B, we observe no significant variation in esterase activity after these treatments compared to the control sample.

### 3.7. J. curcas L Esterase B Shows High Hydrolysis Rates but No Enantiospecificity/Selectivity towards Tested Chiral and Prochiral Substrates

We tested two different substrates to perform an initial assessment of *J. curcas* L. esterase B potential as a biocatalyst: chiral IPG-octanoate and prochiral diethyl phenylmalonate. For the latter, in all tested reaction times (10 min, 30 min, 60 min, and 120 min), we could only detect malonic acid (100% conversion), indicating a high hydrolysis rate towards the short-chain substrate. Enantiomeric excess values could not be calculated because we could not detect the monoethyl form (the chiral compound that can go through racemic resolution by enantiospecific enzymes).

As shown in Table 6, hydrolysis reaction with IPG-octanoate resulted in lower conversion values than those from diethyl phenylmalonate. Still, once again, the enzyme showed no enantiospecificity toward the tested substrate.

## 4. Discussion

*J. curcas* has gained attention from the scientific community due to its high seed oil content, fitted for biodiesel production. Non-toxic *J. curcas* strains’ seeds can be used as an alternative crop for this purpose, leaving press cake as one of the final byproducts. Thus, finding destinations and uses for such a material is an essential topic in the research field of *J. curcas*. This species seed has two esterase activities and one of them, esterase B, was previously characterized as a 30 kDa monomeric protein with an optimum hydrolysis pH of 7.5 and a pI equal to 9.0, in disagreement with the “electrostatic catapult” model for esterases/lipases. To shed light on this apparent discrepancy, as well as to achieve its proper identification, we aimed to study this protein, repeating initial steps as described by [10] and further analyzing its features, mainly regarding its pI and optimum hydrolysis pH, and how they are consistent, or not, with the “electrostatic catapult” model for esterases/lipases [17,18].

Esterase B was initially described as a pI 9.0 protein [10]. Other reports also reported a carboxylesterase within the *J. curcas* seed with similar features such as pI, molecular mass, optimum pH, and temperature [11]. Therefore, our purification step after ethanol differential precipitation was an anion exchange chromatography in a pH 7.5 buffer. In this condition, the protein of interest should have a positive net charge and should not bind to the resin, easily eluting in the flow-through fraction. After assessing esterase activity in all chromatographic fractions and performing protein identification by mass spectrometry analysis, we observed that the homogeneous 30 kDa flow-through protein band was curcin, a known seed protein from *J. curcas*. Curcin is a well-studied 30 kDa basic (pI~9.0) glycoprotein with ribosome-inactivating activity [33] but is less toxic than ricin, an *R. communis* protein.

Instead of eluting in the flow-through, most of the esterase activity was bound to the resin and eluted in fractions enriched in three protein bands corresponding to malate dehydrogenase, lactoylglutathione lyase, and a putative carboxymethylenebutenolidase (belonging to the DLH family). All these are acidic/neutral polypeptides, with pI values ranging from 5.0–7.0. These proteins were previously identified in a proteomic characterization study of the *J. curcas* press cake [7]. Lactoylglutathione lyase and a DLH family protein were thought to be involved in defense and detoxification processes within the seed context. Among these enriched proteins, only DLH is known to have esterase activity, as it also belongs to the α/β hydrolase fold group of enzymes [13,35]. In the 2D SDS PAGE, this protein was identified in a streak of spots (from pH 5.0–6.0), indicating different DLH proteoforms in the *J. curcas* seed with distinct pI values. Identification of the *J. curcas* DLH sequence (GenBank accession number KDP24851.1) allowed for the comparison with other plant DLH proteins. This alignment showed a high identity among them, indicating similar functions within natural contexts. Aside from that, the *J. curcas* DLH theoretical pI and molecular mass of 5.74 kDa and 22.09 kDa, respectively, are close to our experimental data. Active site residues’ recognition showed a cysteine in the nucleophile position within the catalytic triad; its crucial role was corroborated by activity reduction due to specific cysteine alkylation using iodoacetamide.

Enzymes from the DHL group participate in natural and industrial chloroaromatic compound degradation pathways in bacteria [36]. They have distinctive features among the α/β-hydrolase fold family such as esterases/lipases. They have a catalytic triad, but instead of serine as a nucleophile, they possess a cysteine, thought to be necessary for substrate-assisted catalysis. In this model, a functional group within the substrate modifies cysteine, activating it and allowing for proper reactions [37]. This mechanism is thought to aid in substrate specificity and in reducing activity loss due to cysteine oxidation. A study with rice seed disulfide proteome, aimed at detecting thioredoxin-linked reactions in seed germination, identified a DLH as a thioredoxin target; this brings the discussion regarding whether in higher plants, another activity control mechanism would be essential to activate the DLH, rather than the substrate-assisted one [8].

After building a tridimensional model for *J. curcas* DLH, we could assess its electrostatic behavior at different pH values and the protonation state of titratable residues. We observed that around acidic pH, both the active site and catalytic His-161were positively charged, while the surroundings were near neutral. As pH increased, the active site and His-161 became neutral while surroundings turned to a negatively charged state. These data corroborated the esterase/lipase “electrostatic catapult” model, which states that such enzymes show a higher hydrolytic activity at basic pH due to the negative surface potential, repelling one of the final reaction products, namely the carboxylic acid (negatively charged in the neutral and basic pH range) [17,18]. Moreover, His-161 charge state in basic pH allows for proper conditions for the catalytic cycle [38].

The experimental design showed that, within analyzed ranges, the temperature has a more significant impact on esterase activity, but reaction pH also presents an influence. Once more, this basic optimum pH corroborates the “electrostatic catapult” model. *J. curcas* DLH has optimum pH values and a temperature between 9.0-10.0, and ca. 50 °C. Similar features were associated with other plant esterases such as *Cucurbita pepo* [39] and *Avenua fata* [40]. This optimum reaction temperature, along with its molecular mass and monomeric state, is in accordance with previous studies of *J. curcas* seed esterases [10,11], indicating that they all could be the same enzyme. Conversely, both studies concluded that the esterase had a pI of ca. 9.0, which is not in agreement with our data (pI 5.0–6.0). However, this may have occurred due to curcin contamination in these studies, leading to esterase activity results from DLH, while physicochemical features are derived from the curcin contaminant (same molecular mass). Considering we performed two-dimensional electrophoresis followed by protein identification by mass spectrometry, we could differentiate the 30 kDa esterase from curcin based on their different pI values.

Due to the active site and catalytic mechanism resemblance among the α/β hydrolase family members, it is known that some peptidases can hydrolyze small chain esters [37,41]. Thus, confirming that esterase-detected activity did not derive from a proteolytic activity was an essential step in esterase B characterization. Our results showed no peptidase activity within our working fraction and that peptidase inhibitors did not diminish esterase activity. Moreover, EDTA positively affected esterase activity, indicating that divalent cations inhibit *J. curcas* DLH, as supported by our other assays with different ions. Similar effects were also observed for other plant esterases/lipases such as *C. pepo* [39], *Glycine max*, *Oryza sativa* [42], and those in wheat flour [43]. Metal ions can affect esterases/lipases and other α/β hydrolases activity by different mechanisms such as by coordinating with active site residues [44], overall structure alteration by allosteric regulation [44,45], surface potential alteration (a pH-dependent event) [46], and reaction equilibrium dislocation through low-soluble salt production with one of the hydrolysis reaction products, namely the carboxylic acid (mainly described for Ca^2+^ [47]). Whether it is an activation or inhibition scenario depends on each enzyme–metal ion pair and this activity alteration usually represents a gain or loss of enzyme stability. Thus, further experiments should be performed to assess the actual mechanism for *J. curcas* DLH divalent cation inhibition, exploring these distinct possible scenarios both by activity assays and in silico analysis.

Finally, we performed preliminary assays to assess the *J. curcas* DLH potential in hydrolysis reactions of industrial interest, focusing on producing enantiomerically pure compounds. Our results showed that this enzyme was not enantiospecific/selective towards the tested substrates (solketal ester [IPG-octanoate] and diethyl phenyl malonate, a prochiral compound) but it had high conversion rates. For instance, in the same period and conditions for which Amano AK, a *Pseudomonas fluorecens* lipase, had a conversion rate of about 10% (with product enantiomeric excess >99%) when using a racemic solketal ester in a hydrolysis reaction [24], *J. curcas* DLH had conversion values of 50% (but with enantiomeric excess <5%). Therefore, our initial analysis indicates that this catalyst has high hydrolytic activity. Further studies should be done to analyze this enzyme’s use in specific reactions in chemical processes. Additionally, recombinant enzyme production might be interesting to obtain a highly homogeneous bioproduct without contaminants derived from the *J. curcas* seed, such as curcin.

## 5. Conclusions

This work shows that *J. curcas* seed esterase could be useful when short-chain ester hydrolysis is desired. Our data from the two-dimensional electrophoresis approach and structural analysis after comparative modeling indicate that this enzyme is indeed in agreement with the esterases/lipases’ “electrostatic catapult” model and that the previously described pI value was probably from curcin, contaminating the esterase working sample, as these proteins have a similar molecular mass. Aside from that, mass spectrometry analysis identified the enzyme as belonging to the dienelactone hydrolase class, which harbors a cysteine as the nucleophile within the catalytic triad. These data are corroborated by activity inhibition when adding iodoacetamide, an alkylating reagent. Furthermore, activity maintenance after organic solvent incubation (isopropanol and acetonitrile) could be an interesting feature, allowing for the use of these compounds during purification steps such as in reversed-phase chromatography. However, the general use of *J. curcas* DLH is still impaired by its inhibition by divalent cations and its low activity in the presence of organic solvents (isopropanol, acetonitrile, and ethanol). Aside from that, as a final remark, developing new uses for the *J. curcas* press cake and using it as an enzyme source is essential to develop this crop research field, aggregating the value for the biodiesel production with this plant oil and stimulating this research field.

## Figures and Tables

**Table 1:** Ranges of the factors investigated in the experimental design.

Factor	Level				
	−1.41	−1	0	+1	+1.41
pH	7.0	7.3	8.0	8.7	9.0
Temperature (°C)	30	33	40	47	50

**Table 2:** Protein identification after mass spectrometry analysis. Spots are numbered accordingly to Figure 2 and Figure 3.

Spot n°	Protein	Organism	Theoretical Mass (Da)	*p*-Value	Coverage (%)	Peptides	
						N	Sequence
I/1	Curcin precursor	*Jatropha curcas*	32,514.338	2.42 × 10^−224^	24.91	5	QQTLSFTGSYADFLSR EAFGFSYSSHEIPVLR VGGTSYFFNDPESLADAK SSKPADIAKPLVGFIEMVPEAAR SSKPADIAKPLVGFIEM(+15.99)VPEAAR
II/2	Malate dehydrogenase	*Ricinus communis*	36,103.72	8.3 × 10^−149^	22.61	4	LFGVTTLDVVR DDLFNINAGIVK GYVGEDQLGKALEGSDVVIIPAGVPR LNPLVSNLALYDIANTPGVAADVSHINTR
III/3	Lactoylglutathione lyase	*Ricinus communis*	31,547.15	2.1 × 10^−149^	11.79	4	FYTEC(+57.02)FGMK ITSFLDPDGWK GPTPEPLC(+57.02)QVMLR GPTPEPLC(+71.04)QVMLR
IV/4	Putative carboxymethylenebutenolidase	*Arabidopsis thaliana*	25,893.30	2.4 × 10^−59^	12.97	2	ALIPDLYR APIQAHFGELDNFVGFSDVTAAK

**Table 3:** Analysis of variance (ANOVA) results for the developed model, correlating esterase activity to reaction temperature and pH.

ANOVA; R^2^ = 0.82238; (2 Factors with 2 Levels Each) Central Composite, nc = 4, ns = 4, n0 = 2, and Runs = 10)					
Factor	S.S.	Df	M.S.	F	P
(1) Temperature (L)	0.001857	1	0.001857	11.68790	0.014165
Temperature (Q)	0.001155	1	0.001155	7.26803	0.035769
(2) pH (L)	0.000377	1	0.000377	2.37215	0.174446
pH (Q)	0.000558	1	0.000558	3.51329	0.110009
1L by 2L	0.000730	1	0.000730	4.59470	0.075781
Error	0.000953	6	0.000159		
Total SS	0.005367	11			

**Table 4:** Obtained esterase activity values for the conditions analyzed by this experimental design.

Temperature (°C)	pH	Esterase Activity (U/mL)
33 (−1)	7.3 (−1)	0.186869
33 (−1)	8.7 (+1)	0.183168
47 (+1)	7.3 (−1)	0.183168
47 (+1)	8.7 (+1)	0.233503
30 (−1.41)	8 (0)	0.17268
50 (+1.41)	8 (0)	0.225888
40 (0)	7 (−1.41)	0.204545
40 (0)	9 (+1.41)	0.210396
40 (0)	8 (0)	0.202073
40 (0)	8 (0)	0.23057
40 (0)	8 (0)	0.23057
40 (0)	8 (0)	0.227979

**Table 5:** Evaluation of *J. curcas* esterase model and its template.

Assessment	PDB ID: 4ZV9	Model	Model Refined
MolProbity Score	0.98	1.72	0.88
Clash Score	1.37	4.62	0.00
Ramachandran Favoured	97.42%	91.92%	94.44%
Ramachandran Outliers	0.00%	1.52%	0.64%
Rotamer Outliers	0.54%	0.00%	0.00%
QMEAN	0.03	−2.40	−2.39

**Table 6:** Hydrolysis of IPG-octanoate using the 50–80% EtOH fraction in different reaction time points. X indicates conversion and ee indicates enantiomeric excess.

	Time (h)			
	2		5	
	X (%)	ee (%)	X (%)	ee (%)
50–80% EtOH fraction	50	<0.1	70	<0.1

## Data Availability

The data presented in this study are available within the manuscript.
